# Use of virtual reality equipment to assess the manual dexterity of applicants for ophthalmology residency

**DOI:** 10.5935/0004-2749.20200050

**Published:** 2020

**Authors:** Ibraim Viana Vieira, Bruno Nogueira Sakaya, Lucas Valadao de Brito Soares, Wallace Chamon

**Affiliations:** 1 Department of Ophthalmology and Visual Sciences, Escola Paulista de Medicina, Universidade Federal de São Paulo, São Paulo, SP, Brazil

**Keywords:** Motor skills, Virtual reality, Clinical competence, Ophthalmologic surgical procedures/education, Destreza motora, Realidade virtual, Competência clínica, Procedimentos cirúrgicos oftalmológicos/educação

## Abstract

**Purpose:**

To assess the microsurgery dexterity outcomes of two sequential training evaluations using virtual reality technology.

**Methods:**

This was a multicenter cross-sec tional study of all candidates who were accepted as first-year residents at one of six ophthalmology teaching institutions. Residents were subjected to two identical series of standardized, reproducible dexterity tests using virtual reality equipment (Eyesi^®^): “sequence 1” and “sequence 2.” Each sequence consisted of five difficulty levels that were assessed using a proprietary scoring system. The data were tested for normality using the Shapiro-Wilk test. The differences between tests in sequences 1 and 2 were evaluated using the Wilcoxon signed-rank test.

**Results:**

The data did not follow a normal distribution. There were improvements from sequence 1 in all the tests (all p values<0.05). The sum of all scores (total score) improved from sequence 1 (median= 152.50) to sequence 2 (median 256.00; p<0.001). There was no correlation between the delta sequence values and the average scores.

**Conclusion:**

Two sequential training evaluations using virtual reality technology showed relevant improvement in quantifications of microsurgery dexterity. This information should be considered if virtual reality approaches are used for testing purposes, as previous experience may lead to improved test results.

## Abstract (pt)

**Objetivo:**

Avaliar os resultados da destreza da microcirurgia de duas avaliações sequenciais de treinamento usando a tecnologia de realidade virtual.

**Métodos:**

Estudo transversal multicêntrico em que todos os candidatos que foram aceitos como residentes de primeiro ano em uma de seis instituições de ensino de oftalmologia. Os residentes foram submetidos a duas séries idênticas de testes de destreza padronizados e reprodutíveis usando equipamento de realidade virtual (Eyesi^®^): “sequência 1” e “sequência 2”. Cada sequência consistiu em 5 níveis de dificuldade que foram avaliados usando um sistema de pontuação proprietário. Os dados foram testados quanto à normalidade utilizando o teste de Shapiro-Wilk. As diferenças entre os testes nas sequências 1 e 2 foram avaliadas com o teste de Wilcoxon signed-rank.

**Resultados:**

Os dados não seguiram uma distribuição normal. Houve melhora da sequência 1 em todos os testes (todos os valores de p<0,05). A soma de todas as pontuações (pontuação total) melhorou da sequência 1 (mediana= 152,50) para a sequência 2 (mediana= 256,00; p<0.001). Não houve correlação entre os valores da sequência delta e as pontuações médias.

**Conclusão:**

Duas avaliações sequenciais de treinamento utilizando a tecnologia de realidade virtual mostraram melhora relevante nas quantificações da destreza da microcirurgia. Essas informações devem ser consideradas se abordagens de realidade virtual forem utilizadas para fins de teste, pois a experiência prévia pode levar a melhores resultados.

## INTRODUCTION

In recent years, legal and ethical concerns regarding the use of patients for training purposes have led to the development of alternative approaches for the surgical learning process. The surgical learning curve is known to be associated with increased complication rates, worse patient outcomes, increased operating room time, and increased costs^(1-7)^. Therefore, the well-known saying often heard in a number of teaching institutions, “see one, do one, teach one,” is increasingly being considered as unacceptable^(8)^.

Practice with animals has been recommended for the training of ophthalmic surgeons before they begin actual surgical procedures on human patients^(9)^. For the training of phacoemulsification procedures, slaughterhouse porcine eyes are frequently used; however, despite their anatomical resemblance to the human eye, they do not provide a true simulation of the procedure in human patients^(10)^. Notably, virtual reality (VR) technology has evolved rapidly in recent years and has become widespread in the entertainment industry and in teaching environments^(11)^. Simulators have been used in situations where the possibility of errors during training must be minimal due to high human or economic costs. Therefore, they have been increasingly used for training involving invasive medical procedures^(12,13)^.

Currently, VR simulators can be used for the training of retrobulbar injections, phacoemulsification, and vitreoretinal surgeries^(11)^. Among the available models, EyeSi^®^ (Vrmagic^®^, Mannheim, Germany) has been shown to improve surgical skills^(14)^ and shorten the learning curve^(15)^. In addition to the use of VR simulators as training models, the standardized and controlled scenarios in these simulators may be used for the objective assessment of manual dexterity and surgical competence; to the best of our knowledge, there have been no studies in which VR has been used to evaluate manual dexterity as a potential factor for resident selection.

EyeSi^®^ consists of a mannequin head with an artificial eye, as well as a digital operating microscope and a screen connected to a personal computer. When specific probes (included with the system) are inserted inside the artificial eye, the microscope shows them as instruments to simulate distinct tasks in a VR environment. These tasks constitute a sequence of dexterity movements that increase in difficulty as surgeons complete each phase. The tasks begin with simple single-handed exercises that are not directly related to surgeries; when a threshold score is reached, the next level becomes available. With increasing difficulty, the user must use both hands and both feet to complete the tasks that become progressively similar to those in actual surgeries. The process resembles a video game, and a score is provided for each level.

Although VR is frequently used for training in Europe and the USA, there are minimal data regarding its use for the evaluation of dexterity. We presumed that VR could be used to evaluate manual dexterity in novice surgeons and that it could help to select best-fit candidates for residency and fellowship positions. However, we suspected that, owing to a fast learning curve, previous experience with VR may interfere with its use as a testing device. As such, we assessed the results of two sets of EyeSi^®^ dexterity tests of doctors selected as first-year residents in different ophthalmology institutions in Brazil, all of whom had no previous experience with ophthalmologic surgery or VR surgical simulators.

## METHODS

This cross-sectional study was designed and conducted in accordance with the guideline for good clinical practice and was approved by the local ethics committee (UNIFESP no. 0111/2017). The study included all candidates who were accepted as first-year residents at one of six ophthalmology teaching institutions: Escola Paulista de Medicina, Fundação Banco de Olhos de Goiás, Hospital Oftalmológico de Brasília, Santa Casa de São Paulo, Universidade Estadual de São Paulo, and Universidade de São Paulo. None of the residents had started practical training or had previous experience with any ophthalmology VR simulator. Candidates who did not complete all the required tests were excluded from the study.

After enrollment and the provision of written informed consent, candidates were subjected to two series of standardized, reproducible dexterity tests. During these tests, residents manipulated instruments for which tips were inserted into the anterior chamber of an artificial eye.

**Figure 1:**
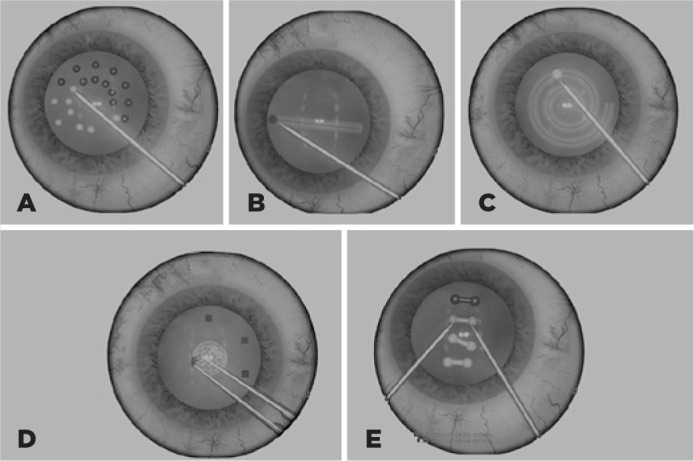
Visual representation of EyeSi® dexterity tests performed in this study. A) Navigation dexterity test in which the tip of the indicator is used to point to spheres until they change colors. B) and C) Antitremor dexterity tests in which an indicator is used to follow the empty region (straight line (#1) and spiral area (#6), respectively). D) Forceps dexterity test in which a VR forceps is used to move virtual squares from outside to inside the central circle. E) Bimanual dexterity test in which two indicators are used to simultaneously touch spheres until they change colors.

The sequence of tests consisted of five levels of increa sing difficulty: navigation (NAV), antitremor #1 (AT1), antitremor #6 (AT6), forceps (FOR), and bimanual (BM) (Figure 1). The ability to differentiate among the levels of ability, known as construct validity, has been previously demonstrated for NAV, AT1, AT6, and FOR^(16,17)^.

In NAV, the tip of the instrument must be sequentially inserted into various adjacent small spheres and held for several seconds until the spheres change color. In AT, the user must “draw” a determined pattern (a horizontal line for AT1 and a spiral line for AT6) at constant speed. In FOR, the user must pinch small squares and bring them to a basket in the center of the eye. In BM, the user must place probes at the ends of a cylinder and hold them steadily for several seconds; if the movement is imprecise, the cylinder may rotate in different axes so that the task becomes more difficult to accomplish^(18)^.

Eyesi^®^ uses a complex proprietary scoring system that gives a total score between 0 and 100 points to each task. If all goals are reached within a task, 100 positive points are given. To perform some tasks, several subgoals must be reached; for example, to draw a spiral in AT6, the user must maintain consistent speed and remain within the intended pattern. Certain movements such as contact with the endothelium or anterior capsule, as well as the use of an open forceps during entry to the eye, are considered mistakes; these lead to a penalty, indicated by negative points. Excessive or unnecessary movements are registered by an odometer and penalized. If the sum is ≤0, the task is scored as 0 points, regardless of whether the user has reached all goals within that task^(18)^.

Before beginning the tests, candidates received instructions on microscope adjustment, positioning, and tasks that they were expected to perform. They also watched instructional videos demonstrating expectations within these tasks. The first sequence of tests was used as an introduction to the platform (sequence 1), because no candidates had previous experience with the simulator or ophthalmic surgeries. Subsequently, on the same day, a second sequence of tests was performed with the same tasks, in order to determine whether the candidates exhibited increased performance (sequence 2). Differences between the personal scores of each test within each sequence (delta sequence) were determined.

The names of the institutions were not attached to the results. A total of 48 medical residents (25 women, 52.1%) were recruited for this study: 8 from Institution A, 6 from Institution B, 7 from Institution C, 6 from Insti tution D, 7 from Institution E, and 14 from Institution F. The data were tested for normality using the Shapiro-Wilk test. The differences between tests in sequences 1 and 2 were evaluated using the Wilcoxon signed-rank test. Stata/IC 15 software was used for all statistical analysis.

## RESULTS

The data did not follow a normal distribution. The re sults of the scores of different tests in sequences 1 and 2 are shown in table 1. Differences were observed between the tests in sequences 1 and 2 (p≤0.0001); there were improvements from sequence 1 in all the tests (NAV, p<0.001; AT1, p<0.001; AT6, p=0.005; FOR, p<0.001; BM, p=0.042).

**Table 1:** Score results from all 48 first-year residents on different tests (EyeSi® simulator) in sequences 1 and 2.

		Average	Median	Minimum	Maximum	Standard deviation	
Sequence 1 Navigation		45.58	45.00	0.00	94.00	27.04	
Antitremor 1		18.06	4.50	0.00	98.00	26.59	
Antitremor 6		16.38	0.00	0.00	88.00	23.66	
Forceps		36.48	33.00	0.00	97.00	33.03	
Bimanual		48.85	52.00	0.00	92.00	23.89	
	Sequence 2						
	Navigation	75.13	81.50	21.00	96.00	18.03	
	Antitremor 1	35.19	36.00	0.00	96.00	27.18	
	Antitremor 6	28.42	23.00	0.00	70.00	26.56	
	Forceps	61.88	76.00	0.00	100.00	33.56	
	Bimanual	56.75	61.00	0.00	94.00	26.62	

Sequence 1 was an introduction to the virtual platform. Later on the same day, sequence 2 was performed to determine whether a performance increase was present.

**Figure 2:**
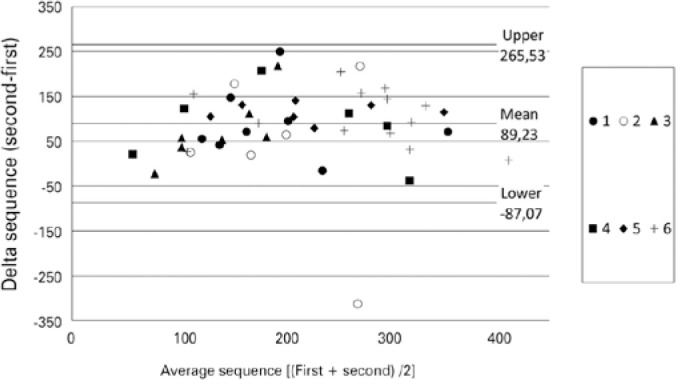
Scatterplot of Total Score results for dexterity tests (EyeSi® simulator) at Sequence 1 and Sequence 2 in subjects that were accepted to be first-year residents in Ophthalmology. Sequence 1 was an introduction to the virtual platform, subsequently, in the same day; Sequence 2 of tests was performed to evaluate a possible performance increase. Each symbol represents one institution.

**Figure 3:**
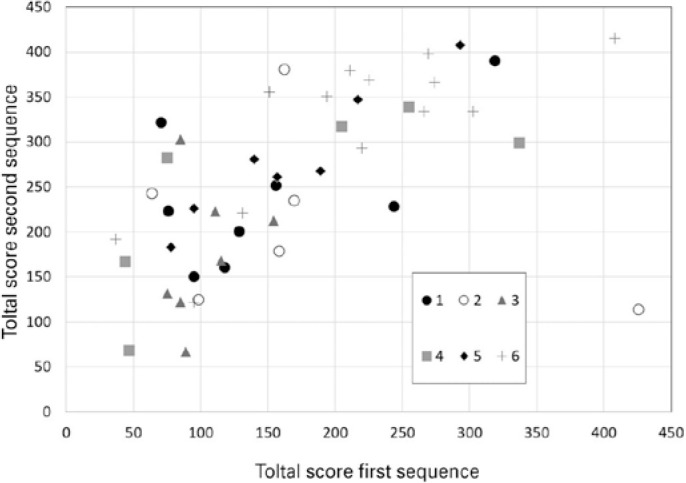
Bland-Altman plot of total score results for dexterity tests (EyeSi® simulator) of first-year residents. Sequence 1 was an introduction to the virtual platform. Later on the same day, sequence 2 was performed to determine whether a performance increase was present. The delta sequence represents the improvement achieved after the initial sequence of tests. Each symbol represents one institution.

The sum of all scores (total score) improved by 103.50 points from sequence 1 (median=152.50) to sequence 2 (median=256.00) (p<0.001), as shown in figure 2. There was no correlation between the delta sequence values and the average scores (Figure 3). Statistical differences between the institutions were detected in sequence 2 (p=0.025).

## DISCUSSION

Previous studies have demonstrated a high variability in motor tasks among novice ophthalmic surgeons in EyeSi^®^, wet-lab environments, and actual cataract surgeries^(10)^. Our study demonstrated differences between the two sequences of tasks performed on the same day, which indicated that a user with minimal experience could perform better than a user who had no prior contact with a VR simulator. Thus, previous experience may represent a bias in terms of the use of VR technology for testing purposes. To minimize this interference, we propose that a practice simulator round is provided before assessment; alternatively, we propose that tests are repeated, and possible performance increases are evaluated.

It has been previously suggested that inconsistency between tests is an indication that there are obstacles in predicting who might achieve the best surgical results^(19)^. A good score in the first attempt may not be fully representative of a trainee’s actual skills, because the trainee may be incapable of maintaining consistent performance. In contrast, a good candidate may fail in the initial attempt and then show rapid improvement with training.

Nevertheless, there is evidence of variations in the learning curves among novice general surgeons. A study of performance proficiency in a laparoscopy simulator showed four learning patterns^(20)^. Group 1 demonstrated surgical proficiency at the beginning of the study; group 2 achieved proficiency after some training; group 3 showed an improvement with training, but did not reach the proficiency threshold; and group 4 underperformed at the beginning and did not improve with training. Although it was impossible to differentiate group 2 from group 3 in the initial tests, there were clear differences between groups 1 and 4; these may represent an opportunity to identify the most capable candidates for residency programs^(20)^.

Furthermore, there is increasing evidence that not all trainees achieve proficiency by the end of the training period during general surgery residency. Some studies suggest that between 5% and 20% of residents do not reach competence, regardless of the use of simulated tasks and continued practice^(20-23)^. Although there have not been similar studies in ophthalmology, we presume that the rates of competency are similar. These estimates are alarming, considering the high costs related to medical training and the general expectation that a licensed surgeon is capable of performing procedures with competence at the end of the training period.

Although manual dexterity is not currently part of the selection process and it remains controversial as to whether such criteria should be used to select candidates, a test that can differentiate dexterity among novice surgeons may enable significant reductions in medical training costs. The cost of operating rooms in the USA is approximately 900-1200 USD per hour^(24)^, and it is estimated that resident training may represent a cumulative annual cost of 53 million USD solely in terms of the added operating time^(18)^. If nontangible costs related to surgical complications are considered, this value is likely to be much higher. It is estimated that medical errors may represent costs of more than 1 billion USD per year in the USA^(25)^.

In addition to the economic cost, there is an important social impact associated with the learning curve. It is well known that novice ophthalmic surgeons exhibit higher complication indexes with worse visual outcomes^(1,6)^; this has direct and indirect social and economic consequences for patients and their families^(25)^. Studies have shown psychological consequences for novice physicians who are typically expected to be “error free”; many experience significant emotional distress, anxiety, guilt, or burnout syndrome during residency training^(26,27)^. A test that could determine the learning capabilities of residency candidates may also help to develop new learning processes that could be used for candidates with different learning profiles. In particular, it is not necessary for all residents to undergo the same surgical training; some residents may need more wet-lab experience or a greater number of supervised surgeries.

This study has some limitations. Notably, it was a cross-sectional study; therefore, we did not evaluate whether the scores were associated with differences in the learning curves among candidates. Future studies should assess whether manual dexterity is associated with the rates of surgical complications among specialties during medical residency. If such an association is demonstrated, dexterity tests may be useful as a se lection tool for ophthalmology residency or eminent sur gical fellowships, and our results may be used as a reference for the establishment of such tests.
